# Performance of serum soluble interleukin-2 receptor as a diagnostic marker for lymphoma in patients with fever

**DOI:** 10.1038/s41598-023-44123-5

**Published:** 2023-11-01

**Authors:** Naoki Kanda, Ryota Yamaguchi, Yu Yamamoto, Masami Matsumura, Shuji Hatakeyama

**Affiliations:** 1 Division of General Medicine, Center for Community Medicine, Jichi Medical University, 3311-1 Yakushiji, Shimotsuke-shi, Tochigi, 329-0498 Japan; 2 Division of Infectious Diseases, Jichi Medical University Hospital, Tochigi, Japan

**Keywords:** Haematological cancer, Tumour biomarkers, Diagnostic markers

## Abstract

There have been few reports on the diagnostic performance of soluble interleukin-2 receptor (sIL-2R) for lymphoma. A cross-sectional study was conducted at a university hospital; all patients who were admitted to the Division of General Internal Medicine and underwent serum sIL-2R testing were included. Patients were divided into two groups based on the presence of fever (≥ 38.0 °C). Among 602 patients, 421 had fever and 76 were diagnosed with lymphoma (48 of the 76 were in the febrile group). In all patients, the area under the receiver operating characteristic curve (AUROC) of sIL-2R for the diagnosis of lymphoma was 0.81 [95% confidence interval (CI), 0.75–0.87]. The AUROC was significantly higher in the febrile group (0.88; 95% CI, 0.81–0.94) than in the afebrile group (0.75; 95% CI, 0.65–0.85). In the febrile group, the sensitivity and specificity were 81.2% and 82.3%, respectively, with an optimal cutoff value of 3,250 U/mL. In the afebrile group, they were 89.3% and 54.9%, respectively, with a cutoff value of 868 U/mL. Serum sIL-2R showed high performance as an adjunctive diagnostic marker for lymphoma, particularly among febrile patients. Different cutoff values should be used for patients with and without fever to maximize diagnostic performance.

## Introduction

Interleukin 2 (IL-2) is a significant cytokine that regulates T-cell responses^1^. It mainly promotes proliferation of both CD4+ and CD8+ T cells. An IL-2 receptor is composed of three subunits. Soluble interleukin-2 receptor (sIL-2R), which is the cleaved chain of the IL-2 receptor alpha (also known as CD25) on the cell membrane due to proteolytic processing, has been recognized as a biomarker of T-cell activation. sIL-2R is considered a tumor-related biomarker of lymphoma. Several studies have reported an association between pretreatment sIL-2R levels and progression free survival in diffuse large B-cell lymphoma and follicular lymphoma^2,3^. sIL-2R has also been evaluated as a prognostic marker after treatment among patients with diffuse large B-cell lymphoma and follicular lymphoma^4,5^.

However, there have been few studies on the performance of sIL-2R as a diagnostic tool for lymphoma^6^. Despite this fact, in Japan, sIL-2R is widely measured in patients suspected to have lymphoma. Moreover, to the best of our knowledge, there have been no reports regarding the impact of sIL-2R in patients with inflammation. The performance of sIL-2R as a diagnostic tool can change in patients with inflammation because of sIL-2R levels due to T-cell activation caused by inflammatory diseases other than lymphoma. This study evaluated the diagnostic performance of sIL-2R for lymphoma in patients with and without fever.

## Patients and methods

### Study design and participants

This cross-sectional study used medical records from the Jichi Medical University Hospital (Tochigi, Japan). All patients (> 15 years old) admitted to the Division of General Internal Medicine between January 2014 and June 2021 and tested for serum sIL-2R anytime from 7 days prior to admission till discharge were included in this study. Hospitalized patients who already had a confirmed diagnosis of lymphoma and were tested for sIL-2R for follow-up purposes (non-diagnostic purposes) were excluded from the study. If a patient had multiple eligible episodes during the study period, only the first episode was included.

We divided the enrolled patients into two groups based on the presence of fever: febrile and afebrile. Patients who had an axillary temperature of 38.0 °C or higher from the onset of illness to the third day of hospitalization were defined as febrile patients. If patients reported having fever prior to admission without information on whether they met the definition of fever, they were classified into the febrile group if the word "high-grade fever" was mentioned in their medical records.

Information extracted from medical records included patient age, sex, history of chronic hemodialysis, and final diagnosis that caused eligible hospitalization. We included only those patients who were histologically confirmed to have lymphoma in the final diagnosis of lymphoma; we reviewed lymphoma subtypes based on histopathological reports. The results of laboratory tests were also collected: white blood cell (WBC) and platelet (PLT) counts as well as levels of hemoglobin, serum sIL-2R, C-reactive protein (CRP), lactate dehydrogenase (LDH), ferritin, and erythrocyte sedimentation rate. The serum concentration of sIL-2R was measured using a chemiluminescent enzyme immunoassay (Lumipulse, Fujirebio Inc., Tokyo, Japan), with a reference range of 154–474 U/mL.

This study was conducted in accordance with the Declaration of Helsinki. The Ethics Committee of Jichi Medical University Hospital approved this study and waived written informed consent because of the retrospective design (approval number 20-081).

### Outcomes

The primary outcome of interest was the performance of sIL-2R in the diagnosis of lymphoma in patients with and without fever. A receiver operating characteristic (ROC) curve was used to determine the diagnostic accuracy and cut-off values of serum sIL-2R levels. The ROC curve of the febrile group was compared with that of the afebrile group. The sensitivity and specificity of the sIL-2R levels for diagnosis were also calculated.

### Statistical analyses

Continuous variables are presented as medians and interquartile ranges (IQRs) and compared using the Wilcoxon signed-rank test. Categorical variables were compared using McNemar’s chi-square test. The sensitivity, specificity, positive likelihood ratio, and negative likelihood ratio of sIL-2R, WBC, PLT, and LDH levels for the diagnosis of lymphoma were determined. The area under the ROC curve (AUROC) was measured to estimate diagnostic performance, and the optimal cutoff values were determined using Youden’s index. The ROC curves of the febrile group were compared with those of the afebrile group using the DeLong test. All *P*-values were two-tailed; *P*-values < 0.05 were considered statistically significant. All statistical analyses were performed using R (version 4.1.1, R Foundation for Statistical Computing, Vienna, Austria).

## Results

A total of 625 patients were included in this study. We excluded 23 patients on chronic hemodialysis from further analysis because serum sIL-2R levels during hemodialysis can be elevated regardless of the presence of lymphoma or other disease^7,8^. Patient characteristics are shown in Table 1. Of the 602 patients, 337 (56%) were men, and 421 (70%) had fever. The degree of fever was not identified in one febrile patient, but a record of “high grade fever” before hospitalization was mentioned.

**Table 1:** Patient characteristics and biomarkers used.

	All (n = 602)			Febrile group (n = 421)			Afebrile group (n = 181)		
	Lymphoma (n = 76)	Non-lymphoma (n = 526)	*P* value	Lymphoma (n = 48)	Non-lymphoma (n = 373)	*P* value	Lymphoma (n = 28)	Non-lymphoma (n = 153)	*P* value
Age, years	72 (60–78)	68 (53–76)	0.036	73 (58–80)	67 (46–76)	0.017	68 (61–78)	70 (63–76)	0.992
Men, n (%)	39 (51)	298 (57)	0.452	28 (58)	204 (55)	0.746	11 (39)	94 (61)	0.048
sIL-2R, U/mL	5170 (1795–12,225)	1145 (666–2060)	< 0.001	8460 (3545–16,650)	1240 (721–2450)	< 0.001	1895 (981–5560)	797 (496–1520)	< 0.001
White blood cells, 10^9^/L	6.0 (4.1–9.0)	7.4 (5.0–11.4)	0.005	5.7 (3.3–8.3)	8.0 (4.6–11.8)	0.003	6.5 (5.0–9.2)	6.8 (5.6–9.6)	0.509
Monocyte count, 10^9^/L	0.6 (0.4–0.9)	0.5 (0.3–0.8)	0.061	0.7 (0.4–1.1)	0.5 (0.3–0.8)	0.027	0.5 (0.4–0.7)	0.6 (0.4–0.8)	0.934
Monocyte percentage, %	11 (7–16)	7 (5–10)	< 0.001	13 (8–17)	7 (5–10)	< 0.001	8 (7–11)	7 (6–10)	0.092
M/L ratio	0.8 (0.5–1.1)	0.5 (0.3–0.9)	0.001	1.0 (0.6–1.3)	0.6 (0.3–1.0)	< 0.001	0.6 (0.3–0.9)	0.5 (0.3–0.8)	0.273
Hemoglobin, g/dL	11.1 (9.0–12.8)	10.8 (9.1–12.6)	0.987	10.3 (8.2–11.3)	10.6 (9.0–12.4)	0.047	12.9 (10.9–14.5)	11.5 (9.6–12.9)	0.022
Platelet count, 10^9^/L	144 (75–240)	227 (134–330)	< 0.001	92 (63–159)	226 (127–332)	< 0.001	232 (201–298)	228 (155–328)	0.721
C-reactive protein, mg/dL	5.7 (1.7–8.8)	5.9 (1.7–13.0)	0.405	7.7 (4.3–12.0)	7.8 (2.8–15.5)	0.950	1.1 (0.4–6.0)	2.5 (0.3–6.1)	0.435
LDH, IU/L	473 (282–870)	263 (194–420)	< 0.001	617 (374–1025)	262 (193–421)	< 0.001	288 (212–486)	264 (194–416)	0.356
Ferritin, ng/mL	583 (202–1839)	417 (186–984)	0.137	961 (398–2,413)	485 (220–1,198)	0.011	176 (70–624)	279 (116–545)	0.213
ESR, mm/h	49 (23–87)	67 (34–100)	0.030	64 (32–89)	71 (42–102)	0.297	32 (10–51)	48 (20–81)	0.100

Data are presented as median (interquartile range) unless otherwise indicated.

*sIL-2R* soluble interleukin-2 receptor, *M/L ratio* monocyte-to-lymphocyte ratio, *LDH* lactate dehydrogenase, *ESR* erythrocyte sedimentation rate.

Of the 602 patients, 76 (13%) were diagnosed with lymphoma (48 in the febrile group and 28 in the afebrile group). The median serum sIL-2R level was significantly higher in patients with lymphoma than in those without lymphoma (5,170 U/mL vs. 1,145 U/mL, *P* < 0.001). In the febrile group, the median sIL-2R levels were significantly higher in patients with lymphoma than in those without lymphoma (8,460 U/mL [IQR, 3,545–16,650 U/mL] and 1,240 U/mL [IQR, 721–2,450 U/mL], respectively). In the afebrile group, similar significant trends with relatively low sIL-2R levels were observed; the median sIL-2R levels were 1,895 U/mL (IQR, 981–5,560 U/mL) in afebrile patients with lymphoma and 797 U/mL (IQR, 496–1,520 U/mL) in those without lymphoma. In the febrile group, WBC and PLT counts were significantly lower, and monocyte percentage, monocyte-to-lymphocyte ratio, and LDH levels were significantly higher in patients with lymphoma than in those without lymphoma, although there was no significant difference between patients with and without lymphoma in the afebrile group (Table 1).

Table 2 shows the final diagnoses of patients who participated in the analysis. A total of 76 patients (12.6%) were diagnosed with lymphoma; 31 had diffuse large B-cell lymphoma (17 belonged to the afebrile group), eight had intravascular lymphoma (all belonged to the febrile group), seven had follicular lymphoma (six belonged to the afebrile group), four had extranodal NK/T-cell lymphoma (all belonged to the febrile group), four had Hodgkin’s lymphoma (three belonged to the febrile group), three had angioimmunoblastic T-cell lymphoma, three had adult T-cell leukemia/lymphoma, and 16 had lymphoma of other or uncertain subtype. Seven patients presented with lymphoma-associated hemophagocytic lymphohistiocytosis. In total, 22 patients, all of whom belonged to the febrile group, were diagnosed with hemophagocytic lymphohistiocytosis (seven lymphoma-associated, four infection-associated, six autoimmune disease-associated, and five other or unknown etiology). Hematologic disorders other than lymphoma and hemophagocytic lymphohistiocytosis included 14 lymphoproliferative disorders (eight Epstein–Barr virus- or methotrexate-associated lymphoproliferative disorders and six idiopathic multicentric Castleman disease [iMCD] or iMCD-TAFRO syndrome; these lymphoproliferative disorders were not histologically proven as lymphoma) and seven hypereosinophilic syndromes.

**Table 2:** Final diagnosis of the study participants.

	All (n = 602)	Febrile group (n = 421)	Afebrile group (n = 181)
Lymphoma	76	48	28
Diffuse large B-cell lymphoma	31	14	17
Intravascular lymphoma	8	8	0
Follicular lymphoma	7	1	6
Extranodal NK/T-cell lymphoma	4	4	0
Hodgkin’s lymphoma	4	3	1
Angioimmunoblastic T-cell lymphoma	3	2	1
Adult T-cell leukemia/lymphoma	3	2	1
Other and uncertain subtype	16	14	2
Hemophagocytic lymphohistiocytosis*	22*	22*	0
Lymphoma-associated*	7*	7*	0
Infection-associated	4	4	0
Autoimmune disease-associated	6	6	0
Other or unknown etiology	5	5	0
Lymphoproliferative disorder	14	10	4
Hypereosinophilic syndrome	7	3	4
Multiple myeloma	5	0	5
Other hematologic disorder	13	6	7
Non-hematologic neoplasm	82	30	52
Infectious diseases	127	111	16
Autoimmune diseases	122	98	24
Drug	18	14	4
Miscellaneous	70	44	26
Not identified	53	42	11

*Seven patients with lymphoma-associated hemophagocytic lymphohistiocytosis are listed (redisplayed).

**Figure 1:**
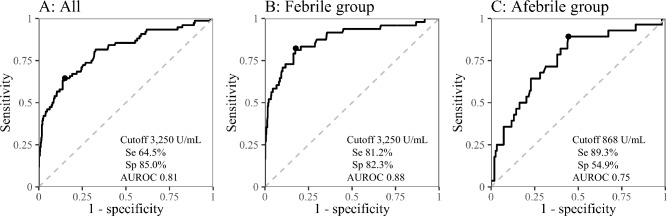
Receiver operating characteristic curve analysis of serum sIL-2R levels for predicting lymphoma in all patients (**A**), febrile patients (**B**), and afebrile patients (**C**). *AUROC* area under the receiver operating characteristic curve, *sIL-2R* soluble interleukin-2 receptor, *Se* sensitivity, *Sp* specificity.

The ROC curves for the prediction of lymphoma by analyzing sIL-2R levels as well as other variables (age, WBC count, monocyte-to-lymphocyte ratio, PLT count, and LDH level) are shown in Fig. 1 and Supplementary Fig. 1, respectively. In all the patients, the AUROC of sIL-2R for the diagnosis of lymphoma was 0.81 [95% confidence interval (CI), 0.75–0.87; *P* < 0.001]. The sensitivity and specificity were 64.5% and 85.0%, respectively, with an optimal cut-off level of 3,250 U/mL. The AUROC (95% CI) was 0.88 (0.81–0.94) in the febrile group (*P* < 0.001) and 0.75 (0.65–0.85) in the afebrile group (*P* < 0.001). The AUROC was significantly higher in the febrile group than in the afebrile group (*P* = 0.044). The sensitivity and specificity were 81.2% and 82.3%, respectively, with an optimal sIL-2R cutoff value of 3,250 U/mL in the febrile patients, and 89.3% and 54.9%, respectively, with an optimal cutoff value of 868 U/mL in afebrile patients. The diagnostic performance (sensitivity, specificity, positive likelihood ratio, and negative likelihood ratio) with different cut-off values is shown in Table 3.

**Table 3:** Diagnostic performance of sIL-2R for lymphoma, stratified by the presence of fever.

Cutoff	Total (n = 602)						Febrile group (n = 421)						Afebrile group (n = 181)					
	Se	Sp	PPV	NPV	LR+	LR−	Se	Sp	PPV	NPV	LR+	LR−	Se	Sp	PPV	NPV	LR+	LR−
500	96.1	16.0	14.2	96.6	1.1	0.2	97.9	12.1	12.5	97.8	1.1	0.2	92.9	25.5	18.6	95.1	1.2	0.3
868^**†**^	93.4	37.6	17.8	97.5	1.5	0.2	95.8	30.6	15.1	98.3	1.4	0.1	89.3	54.9	26.6	96.6	2.0	0.2
1000	85.5	45.2	18.4	95.6	1.6	0.3	93.8	37.8	16.2	97.9	1.5	0.2	71.4	63.4	26.3	92.4	2.0	0.5
2000	72.4	73.4	28.2	94.8	2.7	0.4	87.5	69.2	26.8	97.7	2.8	0.2	46.4	83.7	34.2	89.5	2.8	0.6
3000	64.5	83.3	35.8	94.2	3.9	0.4	81.2	80.7	35.1	97.1	4.2	0.2	35.7	89.5	38.5	88.4	3.4	0.7
3250^**‡**^	64.5	85.0	38.3	94.3	4.3	0.4	81.2	82.3	37.1	97.2	4.6	0.2	35.7	91.5	43.5	88.6	4.2	0.7
4000	56.6	89.0	42.6	93.4	5.1	0.5	72.9	87.4	42.7	96.2	5.8	0.3	28.6	92.8	42.1	87.7	4.0	0.8
5000	50.0	91.4	45.8	92.7	5.8	0.5	64.6	90.6	47.0	95.2	6.9	0.4	25.0	93.5	41.2	87.2	3.8	0.8

*sIL-2R* soluble interleukin-2 receptor, *Se* sensitivity, *Sp* specificity, *PPV* positive predictive value, *NPV* negative predictive value, *LR+* positive likelihood ratio, *LR−* negative likelihood ratio.

^†^The optimal cut-off value in the afebrile group.

^‡^The optimal cut-off value in the total subjects and in the febrile group.

AUROC values (95% CI) of the other variables in all patient cohorts were as follows: age, 0.57 (0.51–0.64); WBC count, 0.60 (0.53–0.67); monocyte-to-lymphocyte ratio, 0.62 (0.55–0.69); PLT count, 0.64 (0.58–0.71); and LDH level, 0.69 (0.63–0.75). The AUROC values of the PLT count and LDH level in the febrile group were significantly greater than those in the afebrile group (Supplementary Fig. 1).

The distribution of serum sIL-2R levels according to the disease category is shown in Table 4. Among patients diagnosed with diseases other than lymphoma, 15% (78/526) had serum sIL-2R levels above 3250 U/mL. In addition to lymphoma, a certain number of patients with hematologic disorders, such as hemophagocytic lymphohistiocytosis, lymphoproliferative disorder, and hypereosinophilic syndrome, tuberculosis, rickettsiosis, and adverse drug reactions showed markedly elevated sIL-2R levels above 5000 U/mL.

**Table 4:** Distribution of serum sIL-2R levels by disease category.

	sIL-2R ≤ 2000 (n = 407)	2000 < sIL-2R ≤ 5000 (n = 112)	5000 < sIL-2R (n = 83)
Lymphoma	21	17	38
Diffuse large B-cell lymphoma	12	7	12
Intravascular lymphoma	1	4	3
Follicular lymphoma	3	3	1
Extranodal NK/T-cell lymphoma	1	0	3
Hodgkin’s lymphoma	1	0	3
Angioimmunoblastic T-cell lymphoma	0	0	3
Adult T-cell leukemia/lymphoma	0	0	3
Other and uncertain subtype	3	3	10
Hemophagocytic lymphohistiocytosis*	7	4	4
Hypereosinophilic syndrome	3	1	3
Lymphoproliferative disorder	3	8	3
Multiple myeloma	5	0	0
Other hematologic disorder	11	1	1
Non-hematologic neoplasm	71	9	2
Infectious diseases	80	33	14
Tuberculosis	5	5	4
Rickettsiosis	0	1	3
Autoimmune disease	100	18	4
Drug	6	9	3
Miscellaneous	64	3	3
Not identified	36	9	8

*sIL-2R* soluble interleukin-2 receptor.

*Non-lymphoma-associated hemophagocytic lymphohistiocytosis only.

## Discussion

This study showed that the diagnostic performance of serum sIL-2R for lymphoma was significantly improved by stratifying the presence of fever, and that different cutoff values should be used for patients with or without fever. The majority of lymphoma subtypes among febrile patients in this study were aggressive lymphomas, including diffuse large B-cell lymphoma, intravascular lymphoma, extranodal NK/T-cell lymphoma, angioimmunoblastic T-cell lymphoma, and adult T-cell leukemia/lymphoma. Although aggressive lymphoma requires prompt diagnosis and treatment, it is sometimes not associated with lymphadenopathy or mass formation and performing biopsies for the confirmation of diagnosis is difficult. Zhang et al. reported that 68% (45/66) of patients with lymphoma who initially presented with fever of unknown origin had aggressive lymphoma and showed poorer performance status, poorer prognosis, and lower complete remission rate than patients with lymphoma without a history of fever of unknown origin^9^. Our results indicate that serum sIL-2R levels may provide important information for estimating the probability of lymphoma and for considering the adoption of further imaging studies, such as positron emission tomography-computed tomography or aggressive biopsy, particularly for febrile patients.

The AUROC of sIL-2R in afebrile patients (0.75) was lower than that in febrile patients (0.88). Murakami et al. evaluated the diagnostic performance of 248 adult patients who were suspected to have lymphoma and had serum sIL-2R tested at a Japanese university hospital. They reported that the AUROC, sensitivity, and specificity were 0.695, 35%, and 93%, respectively, when the sIL-2R cutoff was set at 1,950 U/mL^6^. Their overall results were similar to our results for the afebrile group, mainly because their study included a small fraction of febrile patients (41 of 248 patients, 16%). Studies examining the utility of sIL-2R in the diagnosis of lymphoma are limited; Higashi et al. analyzed the values of sIL-2R in patients who underwent random skin biopsy for the diagnosis of intravascular lymphoma and suggested that high levels of sIL-2R may be one of the diagnostic indicators of the disease^10^.

Although serum sIL-2R levels showed good discriminating power in febrile patients in our study, the role of sIL-2R testing in the diagnosis of lymphoma is complementary, and false positives and negatives should be considered. The levels of sIL-2R are elevated in hematologic diseases other than lymphoma. For example, hemophagocytic lymphohistiocytosis is associated with sIL-2R elevation and is considered a marker for disease activity and prognosis^11–13^. Elevation of sIL-2R levels in patients with hypereosinophilic syndrome has also been reported in previous studies^14,15^. These observations are consistent with our study in which 6 of the 15 patients with non-lymphoma-associated hemophagocytic lymphohistiocytosis and three of the seven patients with hypereosinophilic syndrome had sIL-2R levels above 3250 U/mL.

In addition to hematologic disorders, sIL-2R elevation has been observed in various autoimmune, inflammatory, and infectious diseases, including systemic lupus erythematosus, adult-onset Still’s disease, sarcoidosis, tuberculosis, and rickettsial infections^16–19^. In the present study, the sIL-2R levels in 43% (6/14) of patients with tuberculosis and 75% (3/4) of patients with rickettsial infections were > 3250 U/mL; therefore, we should consider these diseases in addition to lymphomas when sIL-2R levels are elevated in patients with fever of unknown origin. Eighteen patients in this study were diagnosed with having experienced drug reactions; among them, 12 exhibited sIL-2R levels of 2000 U/mL or higher. Several studies have reported elevated sIL-2R levels among patients with drug-induced adverse events^20,21^. Kluge et al. measured cytokine levels in patients who started clozapine and reported that the levels of interleukin-6 and sIL-2R were increased in patients who developed drug-induced fever, suggesting that drug reactions may activate inflammatory cytokines, resulting in the elevation of sIL-2R levels^20^.

In a real-world setting, some patients do not obtain a final diagnosis for a variety of reasons, such as the patients or their households do not wish to undergo invasive examination, or the patient's critically-ill condition does not allow for evaluation. In this study, there were four patients who were strongly suspected to have lymphoma but this was not confirmed histologically; we categorized these patients into the non-lymphoma group (final diagnosis was not identified). However, the diagnostic performance remained approximately the same when they were categorized into the lymphoma group: the AUROC (95% CI) was 0.81 (0.75–0.87) in all patients, 0.88 (0.81–0.94) in the febrile group, and 0.75 (0.65–0.85) in the afebrile group.

We excluded patients on chronic hemodialysis from our study because sIL-2R levels may be elevated by factors other than active disease. The mechanism is thought to involve a combination of factors, including decreased renal clearance and increased production of sIL-2R due to exposure to cytokine elicitors in the dialysate solution^7,8^. A preliminary, unpublished subgroup analysis from our study showed that the median serum sIL-2R level of patients without lymphoma on chronic hemodialysis (n = 23) was significantly higher than that of non-lymphoma patients not on hemodialysis: 3880 U/mL (IQR, 2122–7932 U/mL) vs. 1230 U/mL (IQR, 718–2380 U/mL) in the febrile group and 1720 U/mL (IQR, 1255–1720 U/mL) vs. 797 U/mL (IQR, 496–1520 U/mL) in the afebrile group. In our study, none of the patients with lymphoma underwent chronic hemodialysis. Further independent studies are warranted for patients on hemodialysis to assess the diagnostic performance of serum sIL-2R in lymphoma.

The present study has several limitations. First, this was a single-center study that enrolled patients who were hospitalized at the Division of General Internal Medicine. Therefore, there may have been a bias in the patient population. The participants of this study were predominantly older patients, and it is uncertain whether our results can be generalized to younger patients. Although patients who did not require hospitalization for diagnosis and those who were referred to our hospital with a confirmed diagnosis of lymphoma were not included, the results of our study may provide important insight into estimating the likelihood of lymphoma for a population that is hospitalized for examination of fever. Second, we defined fever as an axillary temperature of ≥ 38.0 °C. The definition of fever can vary across studies, and the degree of fever can be affected by medications, such as antipyretics. Third, this study included patients for whom a final diagnosis could not be obtained even after close examination or for whom further examination was not attempted based on their condition or willingness. Therefore, some cases of lymphoma may be misclassified as non-lymphoma. Fourth, patients diagnosed with lymphoma might have a certain simultaneous complication (e.g., a secondary bacterial infection) that can affect sIL-2R levels, which may have affected the performance of sIL-2R for the diagnosis of lymphoma. Finally, this retrospective study used medical records, which limited the reliability of the information.

In conclusion, this study showed that serum sIL-2R levels are a useful adjunctive diagnostic tool for lymphoma, particularly in febrile patients. To maximize the sensitivity and specificity for lymphoma, cutoff values of serum sIL-2R should be categorized based on the presence or absence of fever. The optimal cutoff levels for patients on hemodialysis remains a subject for future research.

### Supplementary Information

Supplementary Figure 1.

Supplementary Legends.

## Data Availability

The datasets used and/or analyzed during the current study are available from the corresponding author on reasonable request.
