# Supplementary material for: Performance of serum soluble interleukin-2 receptor as a diagnostic marker for lymphoma in patients with fever
Source: Sci Rep. 2023 Nov 1;13:18784. doi: 10.1038/s41598-023-44123-5 (PMC10620379; doi:10.1038/s41598-023-44123-5)
Supplement: Supplementary file 1 — Supplementary Figure 1. [file 41598_2023_44123_MOESM1_ESM.pptx]

## Slide 1
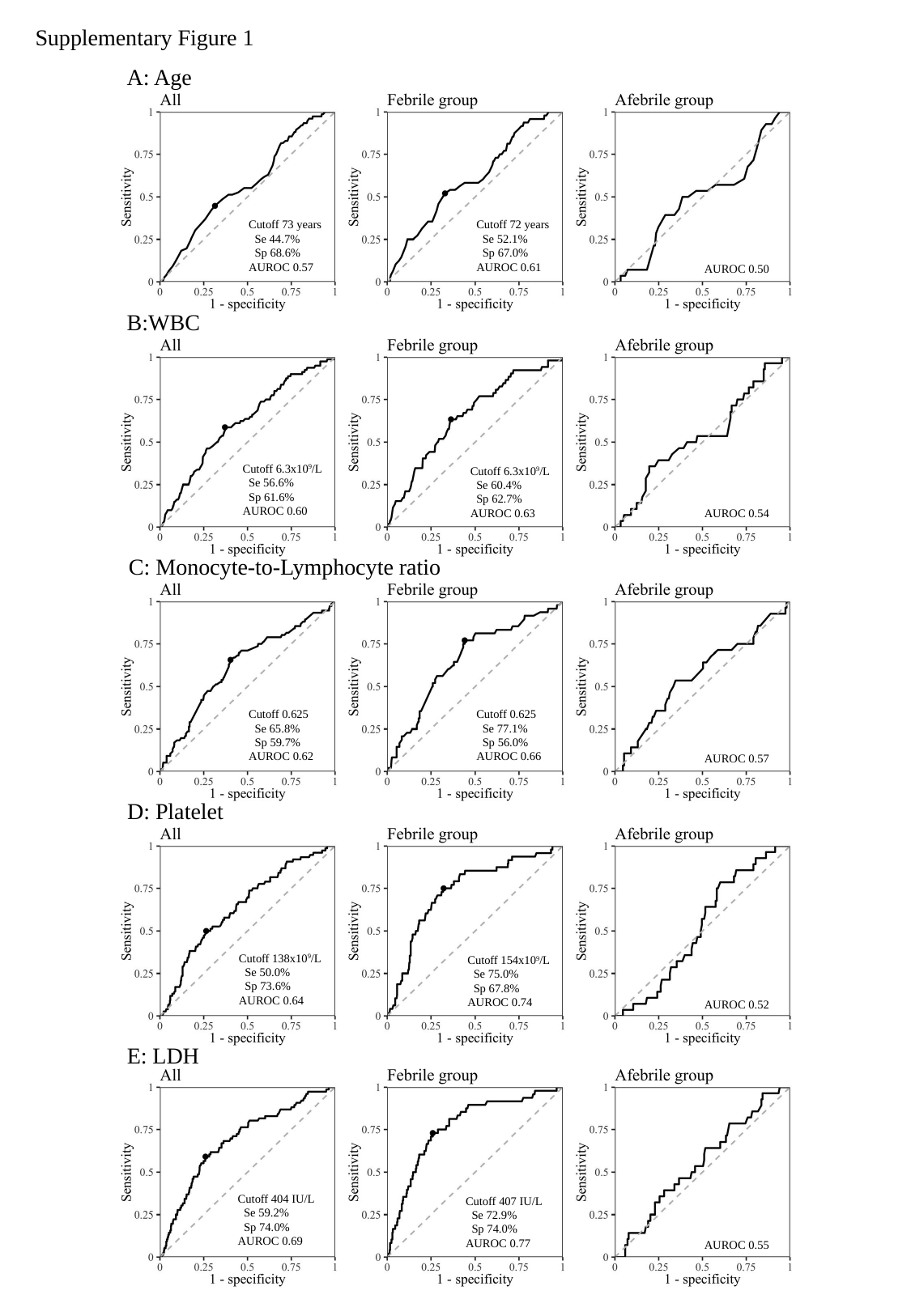

Supplementary Figure 1
A: Age
Cutoff 72 years
 Se 52.1%
 Sp 67.0%
AUROC 0.61
Cutoff 73 years
 Se 44.7%
 Sp 68.6%
AUROC 0.57
AUROC 0.50
B:WBC
Cutoff 6.3x10^9^/L
 Se 56.6%
 Sp 61.6%
AUROC 0.60
Cutoff 6.3x10^9^/L
 Se 60.4%
 Sp 62.7%
AUROC 0.63
AUROC 0.54
C: Monocyte-to-Lymphocyte ratio
Cutoff 0.625
 Se 77.1%
 Sp 56.0%
AUROC 0.66
Cutoff 0.625
 Se 65.8%
 Sp 59.7%
AUROC 0.62
AUROC 0.57
D: Platelet
Cutoff 138x109/L
 Se 50.0%
 Sp 73.6%
AUROC 0.64
Cutoff 154x109/L
 Se 75.0%
 Sp 67.8%
AUROC 0.74
AUROC 0.52
E: LDH
Cutoff 404 IU/L
 Se 59.2%
 Sp 74.0%
AUROC 0.69
Cutoff 407 IU/L
 Se 72.9%
 Sp 74.0%
AUROC 0.77
AUROC 0.55
