# Supplementary material for: Performance of serum soluble interleukin-2 receptor as a diagnostic marker for lymphoma in patients with fever
Source: Sci Rep. 2023 Nov 1;13:18784. doi: 10.1038/s41598-023-44123-5 (PMC10620379; doi:10.1038/s41598-023-44123-5)
Supplement: Supplementary file 2 — Supplementary Legends. [file 41598_2023_44123_MOESM2_ESM.docx]

Supplementary Figure 1. Receiver operating characteristic curve analysis of age and laboratory markers for predicting lymphoma in febrile, and afebrile groups.

AUROC, area under the receiver operating characteristic curve; WBC, white blood cell; LDH, lactate dehydrogenase; sIL-2R, soluble interleukin-2 receptor; Se, sensitivity; Sp, specificity.
